# Diagnosis model of early *Pneumocystis jirovecii* pneumonia based on convolutional neural network: a comparison with traditional PCR diagnostic method

**DOI:** 10.1186/s12890-024-02987-x

**Published:** 2024-04-25

**Authors:** Yingying Li, Hailin Liu, Qingwen Lv, Jun Long

**Affiliations:** 1 Department of Clinical Laboratory, Zhujiang Hospital, Southern Medical University, Guangzhou, China; 2 School of Biomedical Engineering, Southern Medical University, Guangzhou, China; 3 Department of Information, Zhujiang Hospital, Southern Medical University, Guangzhou, China

**Keywords:** Convolutional neural network, *Pneumocystis jirovecii* pneumonia, Diagnostic model, PCR detection

## Abstract

**Background:**

*Pneumocystis jirovecii* pneumonia (PJP) is an interstitial pneumonia caused by *pneumocystis jirovecii* (PJ). The diagnosis of PJP primarily relies on the detection of the pathogen from lower respiratory tract specimens. However, it faces challenges such as difficulty in obtaining specimens and low detection rates. In the clinical diagnosis process, it is necessary to combine clinical symptoms, serological test results, chest Computed tomography (CT) images, molecular biology techniques, and metagenomics next-generation sequencing (mNGS) for comprehensive analysis.

**Purpose:**

This study aims to overcome the limitations of traditional PJP diagnosis methods and develop a non-invasive, efficient, and accurate diagnostic approach for PJP. By using this method, patients can receive early diagnosis and treatment, effectively improving their prognosis.

**Methods:**

We constructed an intelligent diagnostic model for PJP based on the different Convolutional Neural Networks. Firstly, we used the Convolutional Neural Network to extract CT image features from patients. Then, we fused the CT image features with clinical information features using a feature fusion function. Finally, the fused features were input into the classification network to obtain the patient's diagnosis result.

**Results:**

In this study, for the diagnosis of PJP, the accuracy of the traditional PCR diagnostic method is 77.58%, while the mean accuracy of the optimal diagnostic model based on convolutional neural networks is 88.90%.

**Conclusion:**

The accuracy of the diagnostic method proposed in this paper is 11.32% higher than that of the traditional PCR diagnostic method. The method proposed in this paper is an efficient, accurate, and non-invasive early diagnosis approach for PJP.

## Introduction

*Pneumocystis jirovecii* (PJ) is a common opportunistic pathogen causing pulmonary infections and is one of the leading causes of high mortality in immunocompromised patients. *Pneumocystis jirovecii* pneumonia (PJP) occurs in 70–80% of patients with patients with human immunodeficiency virus (HIV) [1]. Over the past decade, the incidence of PJP in patients with HIV has decreased due to the widespread use of antiretroviral therapy. Statistics from the United States on opportunistic infections of PJ in acquired immune deficiency syndrome (AIDS) patients showed a five-fold reduction in incidence per 100,000 person-years between 2000 and 2010 [2]. These findings are supported by research from England, where the number of PJP cases in HIV-infected patients decreased by approximately half during the same period. However, in recent years, there has been an increasing number of non-HIV-infected patients with PJ infections. This includes patients with solid malignancies, solid organ transplant and hematopoietic stem cell transplant recipients, those who have undergone chemotherapy and molecular targeted therapy, patients receiving immunosuppressive therapy due to autoimmune and inflammatory conditions, and individuals with genetic or primary immunodeficiency disorders [3].

Due to the difficulty of culturing PJ in the laboratory, microscopic examination of the pathogen in respiratory specimens has become the gold standard for diagnosing PJP [4]. However, whether cysts and trophozoites of PJ can be observed under the microscope depends on the technical skills and experience of the examining physician, so the level of the observer has a significant impact on the detection rate and sensitivity. Additionally, non-HIV-infected patients with PJ infections typically have lower fungal loads compared to HIV-infected patients [5]. This lower fungal load can result in false-negative microscopic examination results. Currently, PJP diagnosis guidelines recommend a comprehensive analysis of host factors, microbiological evidence, chest CT findings, polymerase chain reaction(PCR), and detection of CD4^+^T cells, lactate dehydrogenase(LDH), and 1,3-β-d-glucan (BDG) in serum for HIV-positive patients with PJP, categorized by severity [6]. However, in clinical practice, PJP has a rapid onset and a poor prognosis. Early diagnosis is largely influenced by the treating physician's experience and judgment. Most non-HIV patients are already at a severe stage when diagnosed [7]. Without timely and effective treatment, the mortality rate can be as high as 30% to 59%, with even higher mortality rates (48% to 70%) among allogeneic hematopoietic stem cell transplant (allo-HSCT) recipients [8]. Therefore, there is an urgent need for an efficient, objective, and convenient method to diagnose PJP.

In recent years, deep learning has made significant progress in the field of medicine and has been shown to outperform traditional machine learning techniques. Convolutional Neural Networks (CNN) is an efficient deep learning method for image recognition, with powerful feature extraction capabilities. They can quickly analyze and process clinical data and medical images to provide diagnostic results. Many studies have reported that deep learning algorithms can improve the accuracy of diagnoses for various diseases, including gastric cancer, thyroid cancer, liver cancer, colorectal cancer, breast cancer, lung cancer, cervical cancer, skin cancer, cataracts, and diabetic retinopathy [9–18]. Recognizing the importance of clinical features and chest CT images in PJP diagnosis, we have developed a novel early diagnostic model for PJP based on CNN and evaluated its diagnostic performance for the first time.

## Materials and methods

**Fig. 1:**
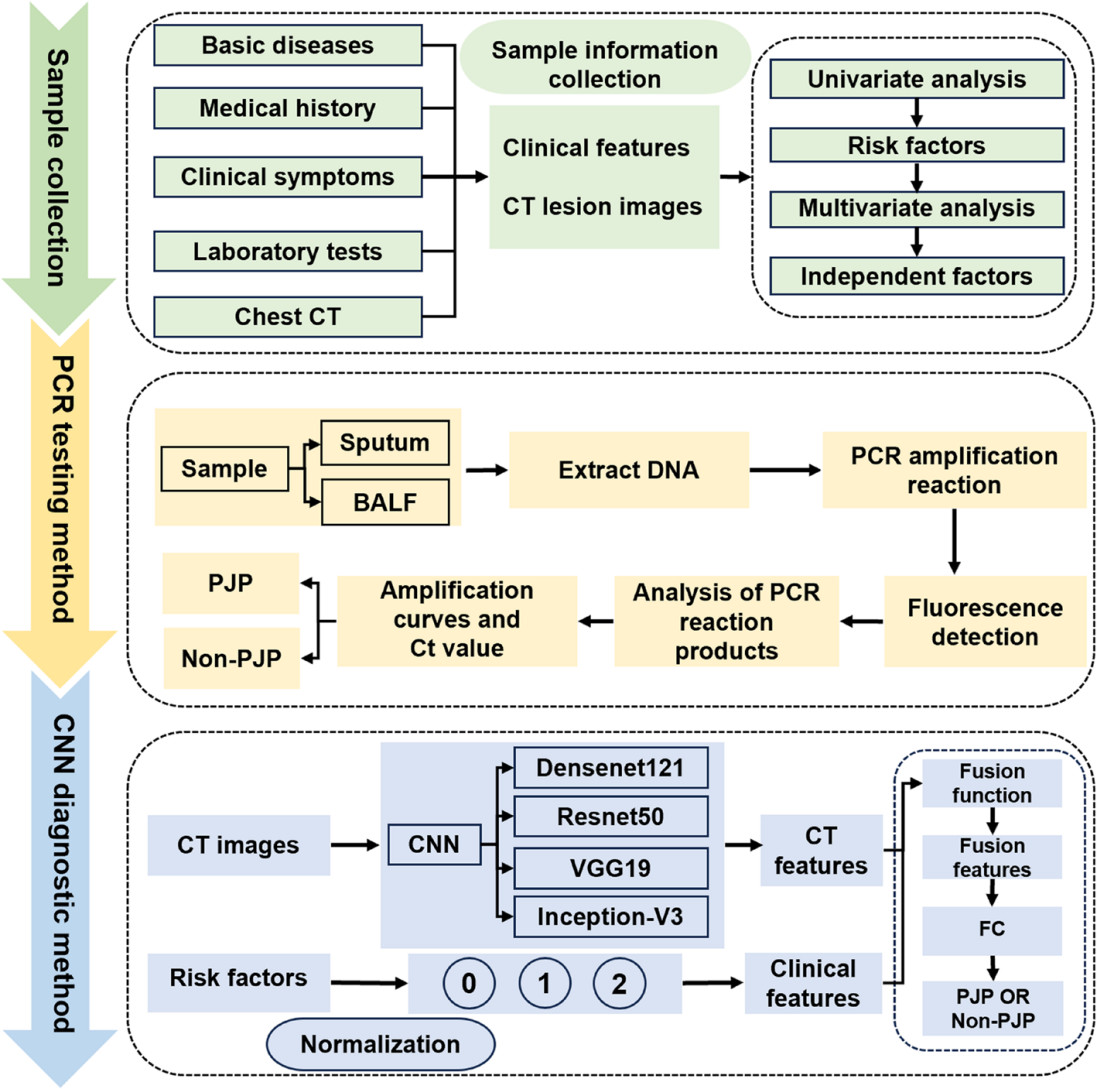
The experimental flowchart of the study

This section introduces the experimental methods for the entire article. Figure 1 shows the experimental flowchart of the study.

Firstly, we will identify all possible diagnostic indicators, clinical features, and chest CT imaging features related to PJP based on the literature and guidelines. Then, from these clinical features, we will select independently significant risk factors with statistical significance.

Next, We are using a Pneumocystis jirovecii DNA extraction kit to extract DNA from sputum and bronchoalveolar lavage fluid samples of both PJP and non-PJP patients in the laboratory. Subsequently, we amplify the target DNA fragments using a PCR thermal cycler. Then, we observe the amplification curve and Ct value of the products in the analysis software provided by the instrument to determine whether the PCR result is positive.

Finally, there is the CNN diagnostic method: first, the patient's CT images are read into CNN to extract CT image features. Then normalize the patient's risk factors to obtain clinical features. Due to the fact that the clinical features of patients are binary variables, we will mark positive clinical features as "1" and negative clinical features as "0". In addition, due to the possibility of missing clinical features in patients, we will mark missing clinical features as "2". Using the feature fusion function, the obtained CT image features are fused with clinical features to obtain the fused features. Finally, the fused features are read into the fully connected layer (FC) structure classification to obtain the final diagnostic result.

### PCR diagnostic method

**Fig. 2:**
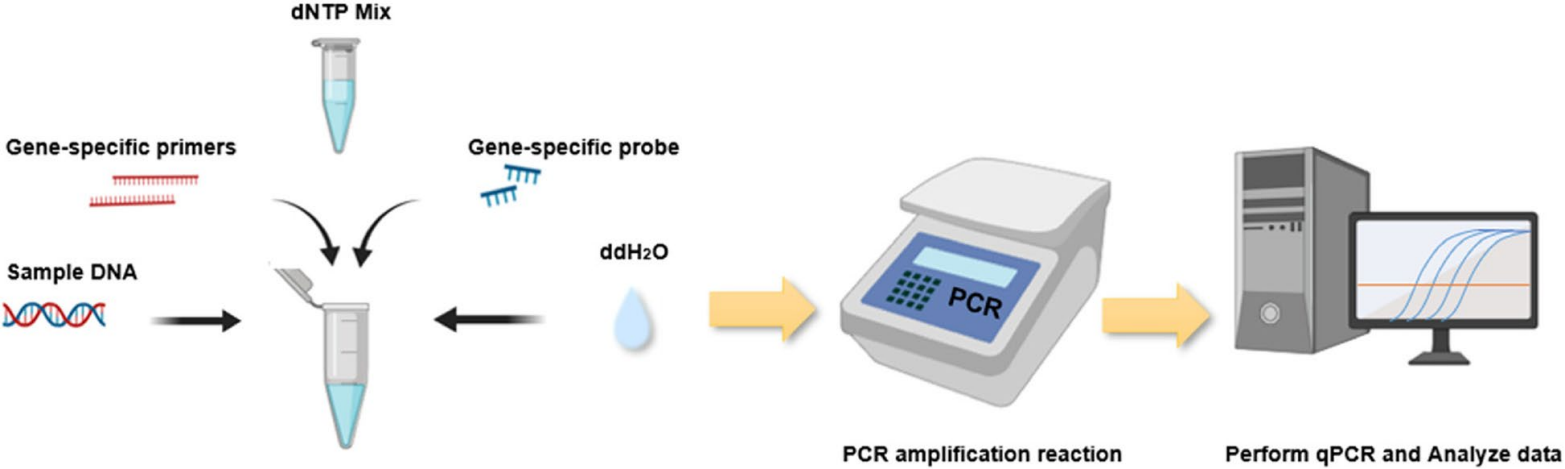
The PCR detection process of PJ DNA

Pulmonary alveolar lavage fluid (BALF) or sputum was centrifuged at 13,000 rpm for 10 min. Sample DNA was extracted using a nucleic acid extraction kit produced by Hangzhou Dilan Biotechnology Co., Ltd., following the manufacturer's instructions. Subsequently, the target gene was amplified using the Dilan Biotechnology PJ nucleic acid detection kit, as shown in Fig. 2. The main reaction system consisted of 4 μL of extracted DNA sample, 4 μL of dNTP Mix, 1 μL of primers, 0.5 μL of probes, and 10.5 μL of distilled water (dd H_2_O). After amplification using a PCR machine, the results were analyzed based on a standard curve. The dNTP Mix is a premixed reagent containing dNTPs, Taq enzyme, UNG enzyme, MgCl_2_, and Buffer.

**Fig. 3:**
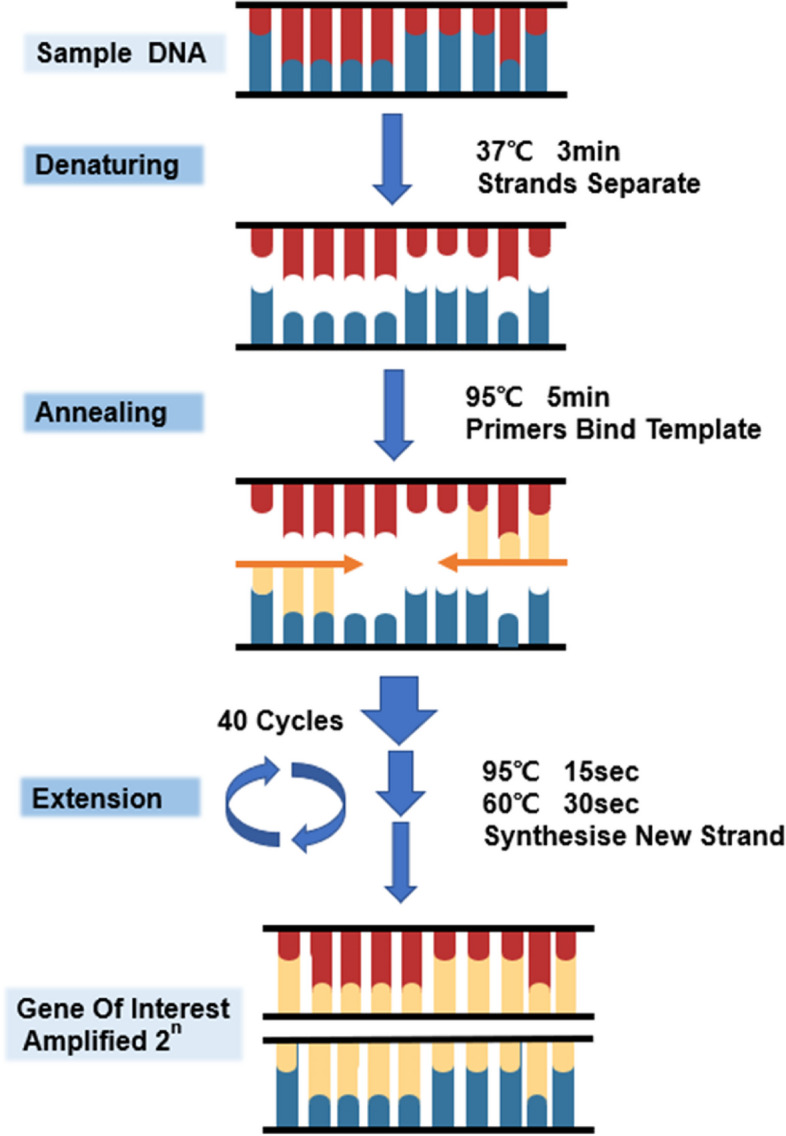
Process of target gene amplification

In this study, the target gene was amplified using a real-time PCR instrument (Thermo Fisher Scientific, Applied Biosystems 7500, USA) under the following conditions: 37 °C for 3 min, 95 °C for 5 min. Then 95 °C for 15 s and 60 °C for 30 s by 40 cycles, as shown in Fig. 3. Subsequently, the Ct values of amplification curves for each sample were analyzed using ABI 7500 software v2.3. Finally, experimental results were interpreted according to the manufacturer's instructions. when the sample Ct values ≤ 36.00 with normal amplification curves and the internal reference Ct value < 40.00, the result was considered positive.

### The CNN method

This paper proposes a method for diagnosing PJP using a CNN as the main framework. This study chose Densenet121 [19], Resnet50 [20], VGG19 [21] and Inception-V3 [22] network in convolutional neural networks as the core model structures. Taking Densenet121 network as an example. The Densenet121 network in the CNN is selected as the main framework. Compared to Traditional CNNs, the Densenet121 network has fewer parameters and is computationally more efficient [23].

Next, provide specific examples to introduce the structure and performance of Densenet121. Densenet121 consists of three dense convolutional blocks [24]. The first advantage of the dense connection module is that it has fewer parameters compared to traditional CNNs because it does not need to re-learn redundant feature maps [25]. Secondly, the way dense block connects features makes the propagation of features and gradients more efficient, making the network easier to train [26]. Each layer of the dense block can directly utilize the gradient of the loss function as well as the initial input information, which is like a form of implicit deep supervision, aiding in training deeper networks [27]. The vanishing gradient problem becomes more likely to occur as the network gets deeper, and the reason for this is the propagation of input and gradient information across many layers [28, 29]. This dense connection is equivalent to directly connecting the input information and loss function to each layer, which can mitigate the effect of the vanishing gradient phenomenon [30].

**Fig. 4:**
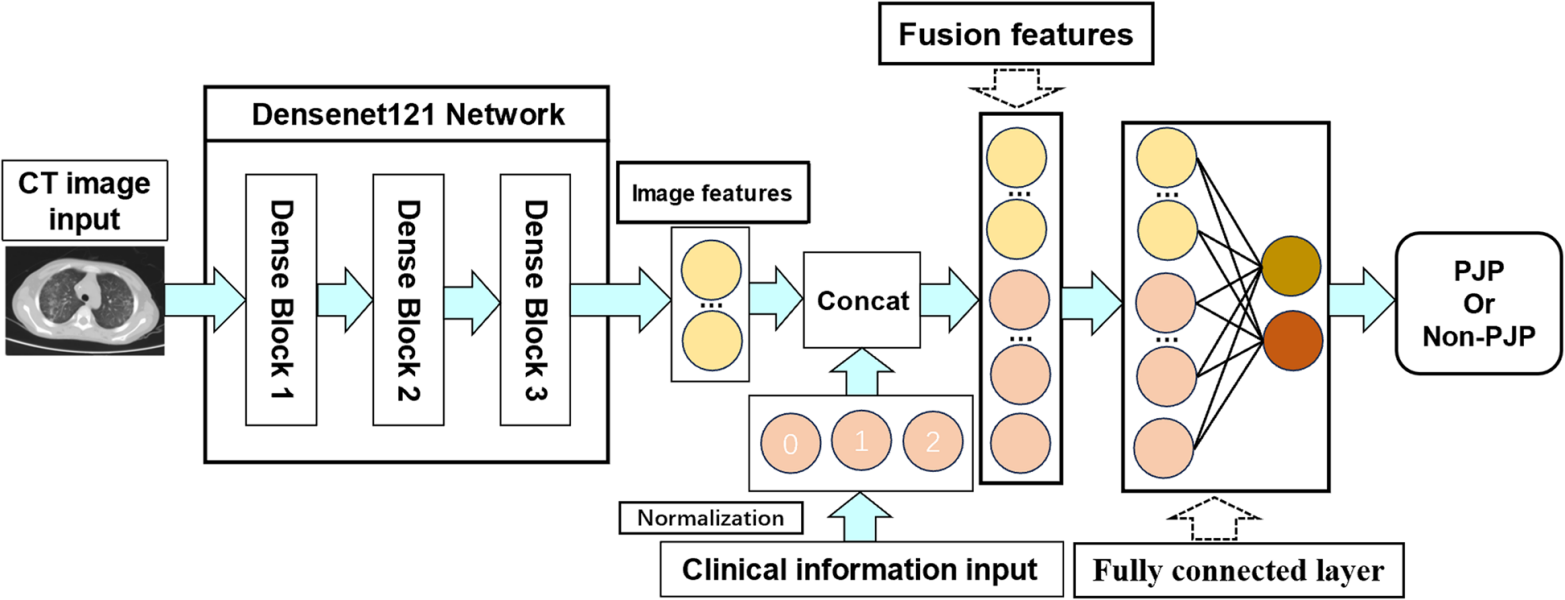
The workflow of the CNN method

Figure 4 illustrates the workflow of this proposed method (Taking Densenet121 network as an example). Firstly, the patient's CT images are input into the DenseNet121 network, which extracts features from the CT images through operations like convolution and pooling [31]. Then, a feature fusion function, Concat, is used to concatenate the patient's CT image features with clinical information features [32]. Finally, the spliced features are input into the last fully connected layer (FC) for classification, then the final diagnostic result is obtained [33].

### Patients selection

The project collected clinical data of PJP at Zhujiang Hospital of Southern Medical University, from August 2022 to August 2023. This study strictly adhered to ethical rules and obtained approval from the hospital's Clinical Medical Center Ethics Committee. Since the study was retrospective, informed consent from patients was not required.

The patient in our study met all of the following inclusion criteria: 1. Age ≥ 18 years; 2. Diagnosed as PJP by two or more clinical physicians based on clinical presentations, laboratory, and imaging examination results; 3. Complete clinical data; 4. Patients can be followed up from admission to discharge or death through the electronic medical record system. At the same time, patients meeting any of the following criteria were excluded: 1. Suspected PJP patients; 2. History of PJP diagnosis before; 3. Missing clinical data, including clinical features, laboratory results, and chest CT imaging results; 4. Pulmonary infections caused by other fungi or pathogens.

To validate the reliability of the model, we used non-PJP patients with other types of pneumonia (Non-PJP) as a control group, matched with PJP patients in a 1:1 ratio, with age (± 5 years) and gender as matching variables.

### Data collection

Review the literature and guidelines related to Pneumocystis jirovecii pneumonia (PJP) [34], find out all possible diagnostic indicators, clinical features, and chest CT imaging characteristics in the diagnosis of PJP, and then collect this clinical information from both PJP patients and non-PJP patients. Categorize the clinical information into clinical manifestations, laboratory test results, chest CT images, diagnostic methods, treatment regimens, and prognosis. All information is entered into electronic medical record forms by one recorder and verified by another. Use the chi-square test to analyze the collected clinical information, select statistically significant indicators, and identify these selected indicators as risk factors for PJP. Further, use multivariate logistic regression analysis on these risk factors to identify more statistically significant indicators, which are then identified as independent risk factors for PJP.

Because this study aims to construct a diagnostic model for early PJP, we only collected data from patients diagnosed with PJP for the first time, with diagnosis confirmed by two clinical physicians. Imaging data was only collected from chest CT images meeting the following criteria: no prior use of any PJP-specific treatment drugs before diagnosis based on clinical symptoms and laboratory test results. The chest CT reports for the matched patient group were independently classified and reviewed by two radiologists.

### CT image preprocessing

In order to eliminate the differences in CT images collected by different medical software and devices and facilitate model training, this study used image standardization and resizing methods to normalize CT images before model training.

All CT images used in the experiment are three channel grayscale images, where each pixel is composed of three values R, G, and B. In the process of image standardization, the average value is subtracted from the R, G, and B values of each channel in each CT image, and then divided by the standard deviation to obtain the standardized image output. Calculate the average and standard deviation based on the R, G, and B values of CT images in the training set. Afterwards, adjust the size of the standardized image to a fixed 256 × 256 pixel size.

## Results

### Statistical analysis

As shown in Table 1 below, chi-square tests revealed 16 clinical factors that were statistically significant, including blood tumors, organ transplantation, immune-related diseases, respiratory failure, chronic kidney disease, hypoalbuminemia, mixed infections, immunosuppressant use, long-term steroid use, surgical history, ground-glass opacity (GGO) on chest CT, mediastinal lymph node enlargement, pleural effusion, lymphocyte reduction, elevated C-reactive protein (CRP), and positive PCR (*P* < 0.05). To ensure the accuracy of the statistical results, laboratory test indicators with some missing data were excluded, including procalcitonin (PCT), LDH, and CD4^+^ T < 200 pcs/μL. Then, multivariable logistic regression analysis was conducted on the clinically significant factors mentioned above, revealing four independent risk factors for PJP, which included blood tumors, long-term steroid use, ground-glass opacity on chest CT, and a positive result in PCR testing. All deep learning methods, statistical analyses, and graphing were performed using IBM SPSS Statistic 25.

**Table 1:** Clinical characteristics and statistical results of PJP and Non-PJP other types of pneumonia

Variables	PJP (*N* = 58)	Non-PJP (*N* = 58)	*P*-value
Basic Diseases			
Hematologic Malignancies	27 (46.55%)	15 (25.86%)	0.003
Organ Transplantation	17 (29.31%)	5 (8.62%)	0.008
Bone Marrow Transplantation	7 (12.07%)	5 (8.62%)	0.762
Immune Disorders	27 (46.55%)	8 (13.79%)	< 0.001
Respiratory Failure	16 (27.59%)	3 (5.17%)	0.002
Hypertension	22 (37.93%)	14 (24.14%)	0.16
Chronic Kidney Disease	26 (44.83%)	14 (24.14%)	0.031
Chronic obstructive pulmonary disease (COPD)	8 (13.79%)	7 (12.07%)	0.782
Low Serum Protein	26 (44.83%)	15 (25.86%)	0.033
Medical History			
Mixed Infection	27 (46.55%)	13 (22.41%)	0.011
Use of Immunosuppressive Agents	42 (72.41%)	20 (34.48%)	< 0.001
Long-term use of corticosteroids	36 (62.07%)	8 (13.79%)	< 0.001
1 year with ≥ 2 hospitalizations	43 (74.14%)	33 (56.90%)	0.078
Mechanical Ventilation	36 (62.07%)	32 (55.17%)	0.572
Surgical History	34 (58.62%)	20 (34.48%)	0.015
Radiation and Chemotherapy	24 (41.38%)	15 (25.86%)	0.115
Clinical Symptoms			
Fever (≥ 38℃)	22 (37.93%)	15 (25.86%)	0.232
Laboratory Tests			
Elevated Neutrophil Count	22 (37.93%)	20 (34.48%)	0.847
Decreased Lymphocyte Count	44 (75.86%)	25 (43.10%)	0.001
Elevated CRP	56 (96.55%)	45 (77.59%)	0.004
Elevated procalcitonin	44 (75.86%)	21 (36.21%)	< 0.001
Elevated LDH	27 (46.55%)	14 (24.14%)	< 0.001
CD4^+^ T < 200 pcs/μL	19 (32.76%)	2 (3.45%)	< 0.001
PJP PCR Positive	51 (87.93%)	19 (32.76%)	< 0.001
Chest CT Imaging			
Ground-glass opacity	30 (51.72%)	9 (15.52%)	< 0.001
Consolidation	16 (27.59%)	11 (18.97%)	0.272
Enlarged mediastinal lymph nodes	23 (39.66%)	12 (20.69%)	0.042
Pleural Effusion	22 (37.93%)	9 (15.52%)	0.011
Nodules	38 (65.52%)	45 (77.59%)	0.217
Fibrous Strands Opacity	32 (55.17%)	25 (43.10%)	0.265

### Results of PCR diagnosis

**Fig. 5:**
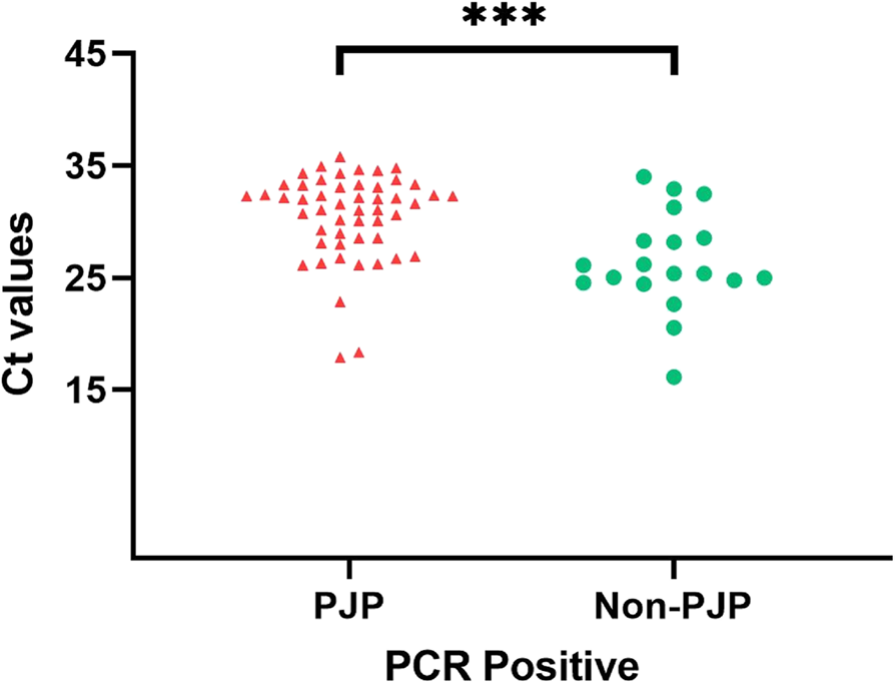
PCR positivity rate in PJP and Non-PJP patients

As shown in Fig. 5, among the 58 cases of PJP, 51 cases (87.93%) had a positive PCR result with a Ct value ≤ 36 and normal amplification curves, while 7 cases (12.07%) had a negative PCR result with a Ct value > 36 or no normal amplification curve. Among the 58 cases of Non-PJP, 19 cases (32.76%) were positive, and 39 cases (67.24%) were negative. In this study, the sensitivity of the standalone PCR test for PJP was 87.93%, with a specificity of 67.24% and an accuracy of 77.58%. There was a significant statistical difference in PCR test results between PJP and Non-PJP (*P* < 0.001).

### Results of the CNN diagnosis

#### Dataset partitioning

**Fig. 6:**
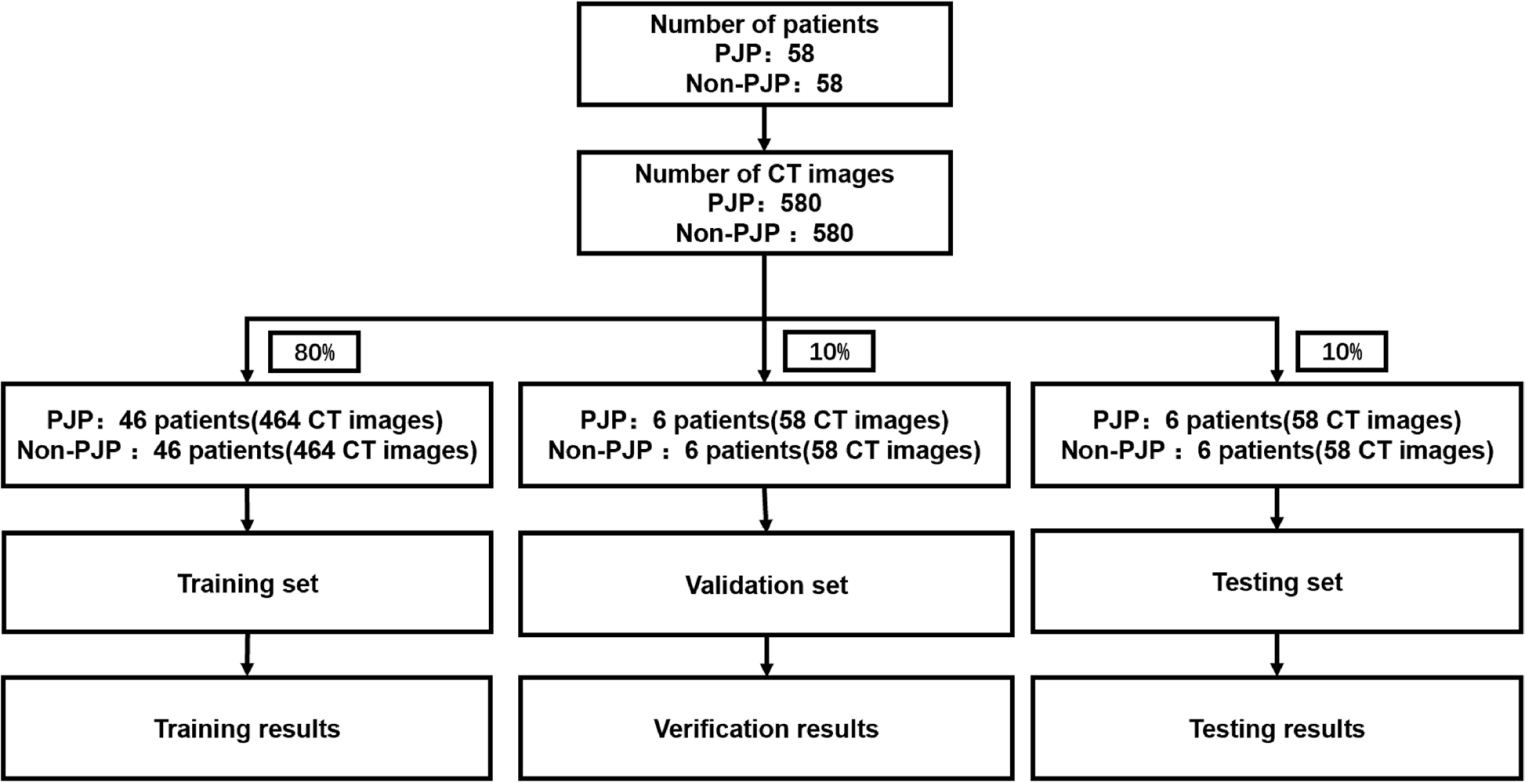
Schematic diagram of data partitioning

In this study, the CT images of PJP and Non-PJP patients were divided into training, validation, and test sets in an 8:1:1 ratio. The specific division is shown in Fig. 6.

The dataset partitioning of this study is independent and random. As shown in Fig. 6, for 58 PJP patients, 46 patients (464 CT images) were used as the training set, 6 patients (58 CT images) were used as the validation set, and 6 patients (58 CT images) were used as the test set. The dataset partitioning rules for Non PJP patients and PJP patients are the same. For 58 Non PJP patients, 46 patients (464 CT images) were used as the training set, 6 patients (58 CT images) were used as the validation set, and 6 patients (58 CT images) were used as the test set.

#### Experimental design

A total of 600 rounds of training were conducted in this study, with the test model saved every 20 rounds, resulting in a total of 30 test models.

During the training process, a total of 600 training epochs were completed. In each epoch, the model received a batch of CT images of patients and corresponding clinical information, and produced model parameters.

The batch size of the model training is 32. The initial learning rate for model training is set to 0.001, which determines the degree of continuous updating and automatic adjustment of model parameters. Generate a set of model parameters every 20 training periods, which are saved and used for model validation on the validation set. Select the parameters with the best diagnostic performance as the final model parameters, and use the test set for the next step of testing. The results of the tests were averaged for each model tested.

#### Statistical analysis

These test models were then used to evaluate the test dataset. In this study, accuracy, recall, precision, specificity, sensitivity, ${F}_{1}$ score and Area Under Curve (AUC) were selected as evaluation metrics for the CNN. All deep learning methods, statistical analyses, and graphing were performed using the Pytorch toolkit and Python 3.7 (Python Software Foundation, www.python.org).

#### Training effect of network model

**Fig. 7:**
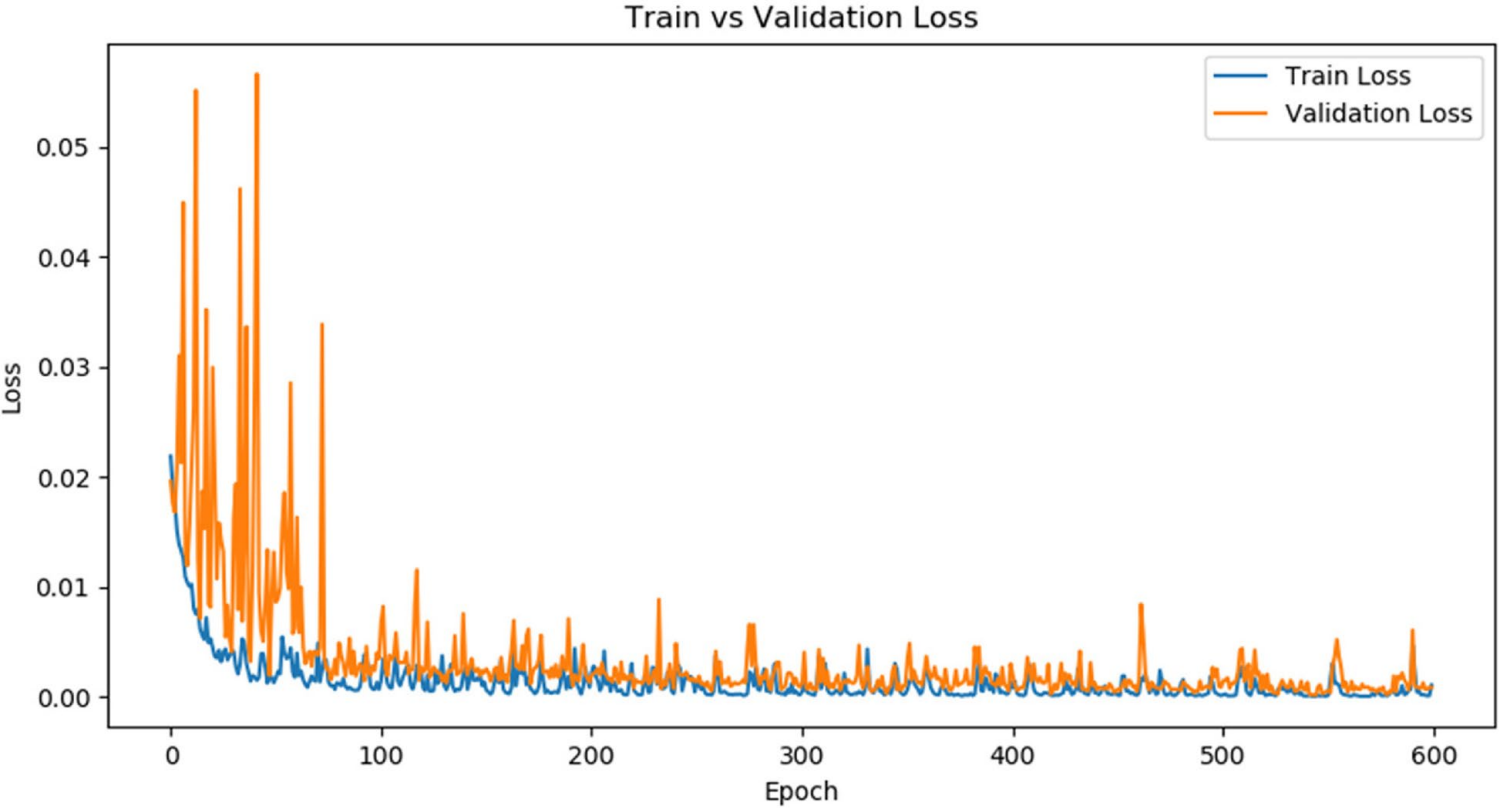
Trends in model training loss and validation loss

**Fig. 8:**
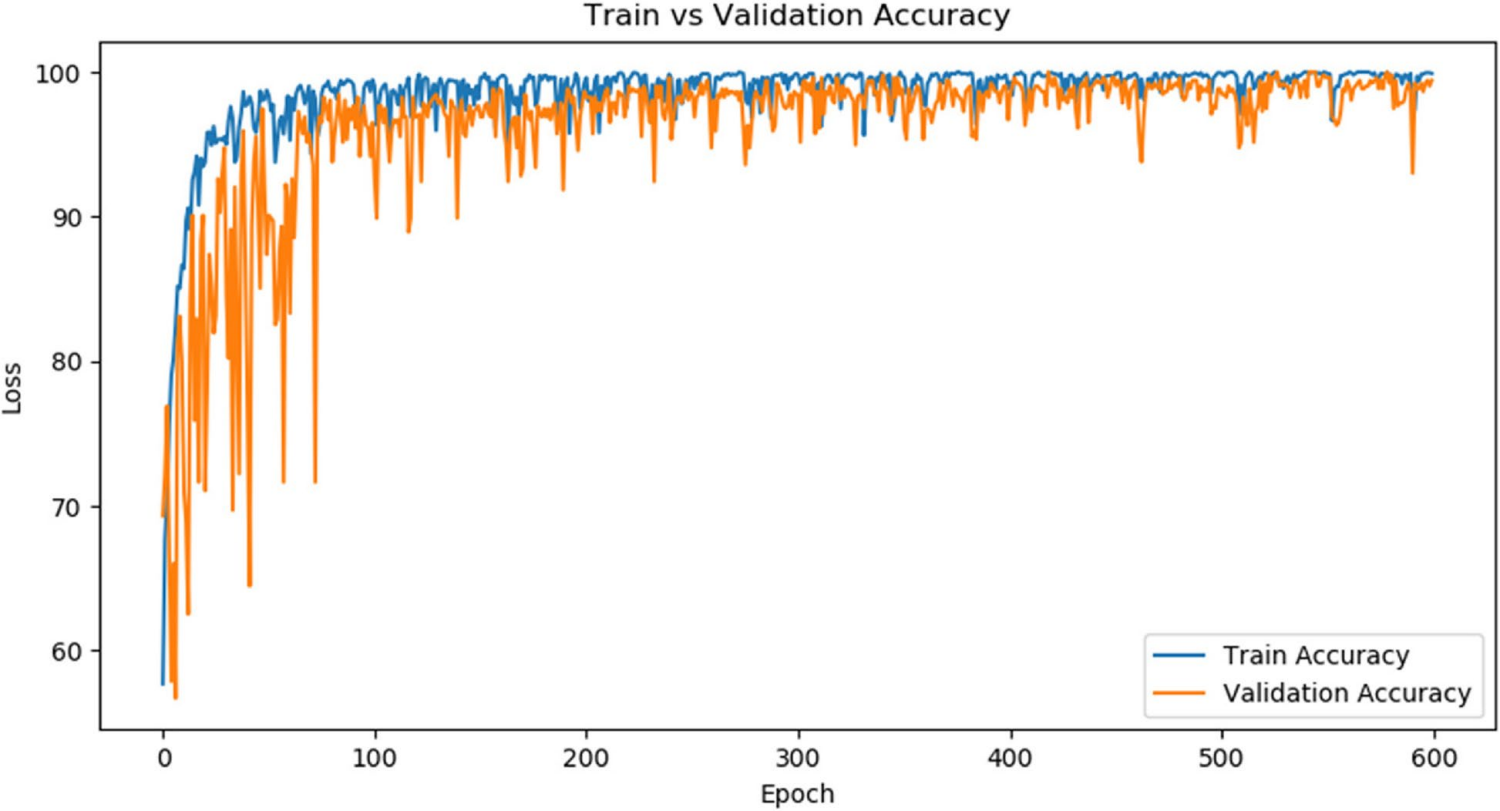
Trends in model training accuracy and validation accuracy

Taking the Densenet121 network model as an example. As shown in Figs. 7 and 8, during the 600 training periods of the model, the training accuracy and validation accuracy of the model gradually improved, while the training loss and validation loss gradually decreased. The accuracy and training loss curves eventually tend to stabilize. No obvious overfitting was observed.

#### Experimental results

**Fig. 9:**
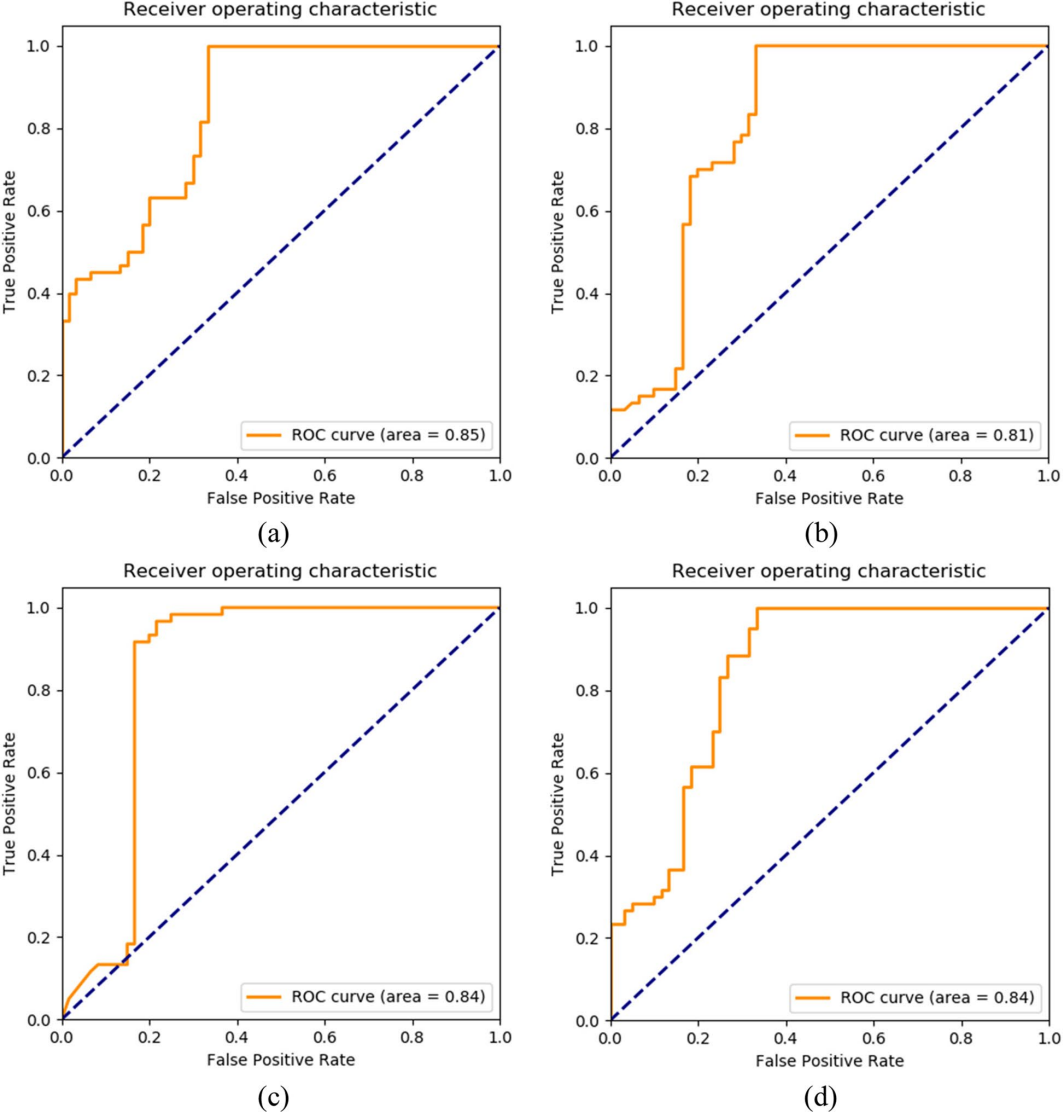
ROC curves for the models: Graphs (**a**) to (**d**) show the ROC curves of Resnet50, VGG19, Inception-V3, Densenet121, respectively

First, input 16 risk factors that have undergone univariate analysis, including the patient's CT image features, as classification criteria into the network. The specific testing results of the top-performing 4 out of 30 test models are shown in Table 2. The ROC curves for these four test models are shown in Fig. 9.

**Table 2:** Testing results of models using 16 risk factors as diagnostic indicators

Models	Accuracy	Recall	Precision	Specificity	Sensitivity	${F}_{1}$	AUC
Resnet50	81.30%	0.93	78.30%	0.73	0.93	0.76	0.85
VGG19	83.50%	0.97	79.50%	0.79	0.97	0.81	0.81
Inception-V3	80.60%	0.95	76.40%	0.74	0.95	0.78	0.84
Densenet121	84.50%	0.97	78.00%	0.73	0.97	0.86	0.84

**Fig. 10:**
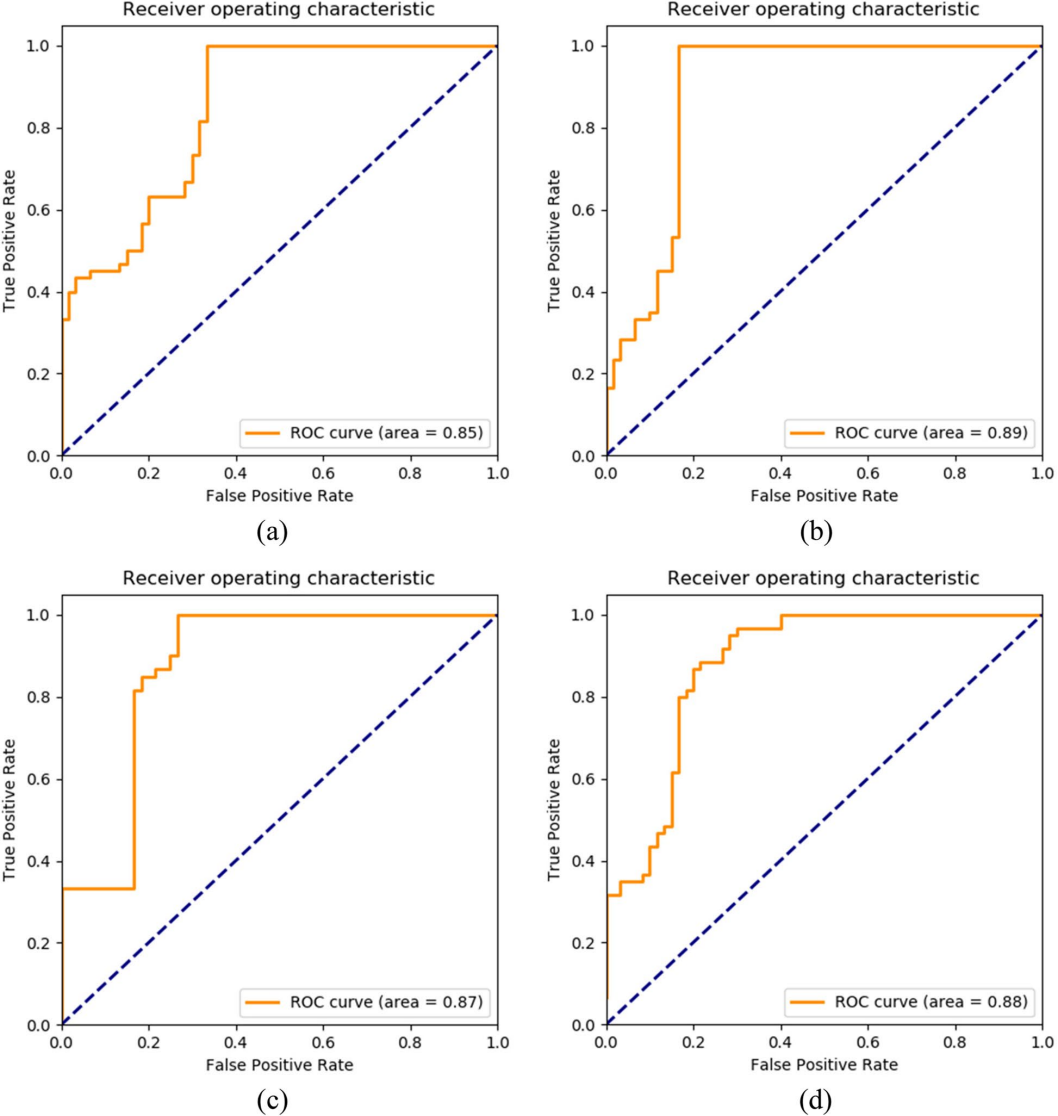
ROC curves for the models: Graphs (**a**) to (**d**) show the ROC curves of Resnet50, VGG19, Inception-V3, Densenet121, respectively

The four independent risk factors obtained through multivariate binary logistic regression analysis were used as classification criteria and input into the network to obtain four test models, with their corresponding AUC curves plotted. The high-performing test models obtained from the 4 independent risk factors are shown in Table 3, and their ROC curves are displayed in Fig. 10.

**Table 3:** Testing results of models using 4 independent risk factors as diagnostic indicators

Models	Accuracy	Recall	Precision	Specificity	Sensitivity	${F}_{1}$	AUC
Resnet50	85.60%	0.93	86.40%	0.73	0.93	0.76	0.85
VGG19	86.20%	0.99	82.30%	0.80	0.99	0.86	0.89
Inception-V3	84.50%	0.96	81.50%	0.74	0.96	0.77	0.87
Densenet121	88.90%	0.98	82.90%	0.80	0.98	0.90	0.88

## Discussion

Based on the above research, the sensitivity of the standalone PCR test for PJP was 87.93%, with a specificity of 67.24% and an accuracy of 77.58%. However, the optimal early PJP diagnostic model Densenet121 based on convolutional neural networks had a sensitivity of 98%, a specificity of 80% and an accuracy of 88.90%. The diagnostic model constructed by combining clinical features and chest CT images significantly improves diagnostic accuracy compared to a standalone PCR test report. In clinical practice, inputting a patient's clinical information and lung CT images into the model proposed in this study can efficiently and rapidly predict the risk of PJP infection. Timely clinical intervention can be taken for high-risk patients to reduce complications and lower mortality.

Although the current gold standard for diagnosing PJP is to detect cysts or trophozoites in lower respiratory tract specimens through traditional staining or immunofluorescence staining methods, with the latter having higher sensitivity than traditional staining, in actual clinical practice, due to cost considerations, immunofluorescence staining is rarely used, and most cases employ traditional staining methods. Therefore, the observation of pathogens through traditional staining methods often serves as the basis for diagnosis, despite the low sensitivity and cumbersome operation of traditional staining methods, and whether pathogens can be found depends on the observer's experience. In addition, non-HIV patients are prone to false negatives due to low fungal loads. Among the 58 PJP patients in this experiment, two experienced senior laboratory physicians observed only 3 positive cases under a microscope using traditional staining methods, further confirming the low sensitivity of traditional staining methods. In recent years, bronchoalveolar lavage fluid combined with metagenomic next-generation sequencing (mNGS) has been used in clinical practice due to its rapidity and high detection rate. However, the high cost of mNGS and its inability to distinguish colonization from infection can lead to unnecessary overtreatment based solely on mNGS results. Moreover, interference from human DNA, microbial loads, and incomplete fungal databases are all shortcomings of mNGS. The PCR mentioned in this study is a commonly used PJP detection method in clinical practice. It not only has high sensitivity, capable of detecting very low levels of fungal loads undetectable by immunofluorescence staining, and can essentially rule out PJP based on negative predictive values, but it is also much cheaper than mNGS. However, PCR has relatively low specificity, so standalone PCR test results also have limitations in distinguishing colonization from infection.

Given the current state of PJP diagnosis, we will identify all possible diagnostic indicators, clinical features, and chest CT imaging features related to PJP based on the literature and guidelines. Then, from these clinical features, we will select independently significant risk factors with statistical significance. We will build an early diagnosis model for PJP by combining PCR test results with clinically significant features and lung CT imaging. Compared to standalone PCR testing, this model showed an increase in sensitivity, specificity, and accuracy by 10.07%, 12.76%, and 11.32% respectively. Additionally, colonization and infection of PJP also depend on the patient's own immune status. In our model, clinical features are closely related to this condition, demonstrating that our model provides a more comprehensive and reliable diagnosis compared to standalone PCR analysis.

However, it is important to note that this study has limitations, including limited sample size and the lack of validation through animal model experiments. Although the preliminary results are promising, the lack of data may affect the diagnostic performance of the model. We believe that incorporating more CT images and a larger volume of clinical information from patients can improve the model's diagnostic efficacy. Although this paper used statistical methods to identify patient risk factors as diagnostic indicators for the neural network model, clinical physicians need to comprehensively consider the patient's clinical presentation in the diagnostic process, rather than solely focusing on statistically significant risk factors. Therefore, in practical applications, this method can serve as an auxiliary diagnostic tool for clinicians in disease assessment.

As medical research continues to advance, treatment methods evolve, algorithmic capabilities improve, and medical diagnostic technologies enhance, the future holds promise for the integration of artificial intelligence techniques with clinical data to offer new diagnostic and treatment strategies for PJP patients in clinical practice.

## Conclusion

This article proposes an early diagnosis method for PJP based on CNN. Compared with independent PCR methods, the accuracy of CNN-based PJP diagnostic methods is 11.32% higher than that of PCR detection. This study overcomes the limitations of traditional PJP diagnostic methods and develops a non-invasive, efficient, and accurate PJP diagnostic method. By using this method, PJP patients can receive early diagnosis and treatment, effectively improving prognosis.

## Data Availability

No datasets were generated or analysed during the current study.

## Abbreviations

PJ: *Pneumocystis jirovecii*
PJP: *Pneumocystis jirovecii* Pneumonia
CT: Computed tomography
mNGS: Metagenomics next-generation sequencing
HIV: Human immunodeficiency virus
AIDS: Acquired immune deficiency syndrome
PCR: Polymerase chain reaction
LDH: Lactate dehydrogenase
BGD: 1,3-β-D-glucan
allo-HSCT: Allogeneic hematopoietic stem cell transplant
CNN: Convolutional neural networks
dd H_2_O: Distilled water
GGO: Ground-glass opacity
CRP: C-reactive protein
PCT: Procalcitonin
AUC: Area Under Curve
